# Deficiency of fibroblast growth factor 21 aggravates obesity-induced atrophic responses in skeletal muscle

**DOI:** 10.1186/s12950-019-0221-3

**Published:** 2019-07-04

**Authors:** Chu-Sook Kim, Yeonsoo Joe, Hye-Seon Choi, Sung Hoon Back, Jeong Woo Park, Hun Taeg Chung, Eun Roh, Min-Seon Kim, Tae Youl Ha, Rina Yu

**Affiliations:** 1 Department of Food Science and Nutrition, University of Ulsan, Ulsan, 44610 South Korea; 2 Department of Biological Science, University of Ulsan, Ulsan, 44610 South Korea; 3 Appetite Regulation Laboratory, Asan Institute for Life Sciences, University of Ulsan College of Medicine, Seoul, 05505 South Korea; 4 Division of Endocrinology and Metabolism, Department of Internal Medicine, University of Ulsan College of Medicine, Seoul, 05505 South Korea; 5 Research Group of Nutrition and Metabolic System, Korea Food Research Institute, Wanju, 55365 South Korea

**Keywords:** Obesity, Inflammation, Muscle, Atrophy, Fibroblast growth factor 21

## Abstract

**Background:**

Obesity-induced skeletal muscle inflammation is a major contributor of skeletal muscle loss/atrophy and is implicated in metabolic complications such as insulin resistance. Fibroblast growth factor 21 (FGF21) is known to be an important metabolic regulator with anti-inflammatory properties. However, the effect of FGF21 on skeletal muscle atrophy is unclear. In this study, we investigated the effect of FGF21 deficiency on obesity-induced skeletal muscle inflammation and atrophy in mice.

**Results:**

The expression of atrophic factors (MuRF1 and Atrogin-1) was upregulated at the mRNA and/or protein levels in the skeletal muscle of FGF21-deficient obese mice compared with wild type obese control mice. This was accompanied by an increase in levels of inflammatory cytokines (TNFα and MCP-1) and a reduction in AMPK phosphorylation. FGF21 treatment markedly suppressed TNFα-mediated inflammatory and atrophic responses in cultured myotubes, and the actions of FGF21 were blunted by the AMPK inhibitor compound C.

**Conclusion:**

These findings suggest that FGF21 deficiency aggravates obesity-induced inflammation and atrophic responses in the skeletal muscle of obese mice, and FGF21 may protect inflammation-mediated atrophy through the AMPK pathway.

## Background

Obesity is closely associated with loss/atrophy of skeletal muscle mass referred to as sarcopenia, which contributes to frailty/physical disability [1, 2] and metabolic complications such as insulin resistance and type 2 diabetes [3]. Obesity-induced skeletal muscle inflammation, characterized by increased levels of inflammatory cytokines such as tumor necrosis factor α (TNFα) and interleukin-6 (IL-6), promotes an imbalance in muscle protein synthesis and degradation leading to muscle atrophy [4]. In particular, the ubiquitin-proteasome system, a major route of protein degradation, is known to be critical for muscle wasting: the system degrades muscle proteins into small peptides including muscle atrophy F-box (MAFbx, called atrogin-1) and muscle RING finger 1 (MuRF1) through adenosine triphosphate-dependent enzymatic reactions [5–7]. TNFα is known to directly induce the ubiquitin-proteasome pathway, through activation of nuclear factor-kappa B (NF-κB) in the skeletal muscle [8, 9]. However, the molecules involved in obesity-induced skeletal muscle atrophy remain elusive.

Fibroblast growth factor 21 (FGF21) is a member of the FGF family with many beneficial activities [10]. FGF21 functions as an endocrine hormone with anti-inflammatory, anti-diabetic, and anti-obesity effects and is produced in peripheral tissues (e.g., liver, white and brown adipose tissues, skeletal muscle, and pancreas) [11]. Exocrine FGF21 action is largely mediated via liver-secreted FGF21 [12], while adipose-derived FGF21 acts in an autocrine fashion [13]. FGF21 promotes weight loss through an increase in fatty acid oxidation and decreases triglyceridemia and glycemia by improving insulin sensitivity in diet-induced obese mice [14, 15]. Transgenic mice overexpressing FGF21 in the liver are protected against diet-induced obesity [10], and FGF21 pharmacotherapy in diabetic and obese mice rapidly improves metabolic abnormalities [15, 16]. In skeletal muscle, FGF21 is secreted in response to the activation of cellular stress pathways, such as autophagy impairment and/or mitochondrial dysfunction [17, 18]. FGF21 deficiency leads to impaired glucose tolerance, elevated blood insulin, and fatty liver development [19, 20]. The biological activity of FGF21 is dependent on binding to its receptors (FGFR1 and β-Klotho). Although FGF21 is considered as a therapeutic target in obesity-related metabolic disorders, the potential of FGF21 to control the atrophy of skeletal muscle in obese conditions is unclear.

In this study, we demonstrate that deficiency of FGF21 aggravates obesity-induced atrophic responses and inflammation in skeletal muscle of mice fed an HFD. Also, FGF21 treatment protects TNFα-induced atrophic responses in muscle cells, and this was reverted by an AMP-activated protein kinase (AMPK) inhibitor.

## Materials and methods

### Cell culture and treatment

The murine myoblast cell line C2C12 (5 × 10^5^ cells/mL) was grown at 37 °C in 5% CO_2_ in DMEM (Life Technologies, Grand Island, NY, USA) containing 10% FBS (Life Technologies) and 1% penicillin-streptomycin (Life Technologies). At a confluence of 95–100%, the medium was replaced with differentiation medium [DMEM plus 2% horse serum (Life Technologies)], which was changed after 2 days. To examine the effects of FGF21 on TNFα-induced muscle atrophy, differentiated myotubes were treated for 24 h with 100 ng/mL of TNFα (Pepro Tech), rmFGF21 (Creative Biomart, Shirley, NY, USA), or both drugs in combination. To examine the link between the effects of FGF21 on TNFα-induced muscle atrophy and AMPK, C2C12 myotubes were treated with rmFGF21, 20 μM compound C (AMPK inhibitor, Sigma), and/or 0.5 mM 5-aminoimidazole-4-carboxamide ribonucleotide (AICAR, AMPK activator, Sigma).

### Animal experiment

Whole-body FGF21-deficient mice (FGF21 knockout/KO) on a C57BL/6 background were purchased from the Jackson Laboratory (Bar Harbor, ME, USA) and bred in a specific pathogen-free animal facility at the University of Ulsan. Male FGF21-deficient mice and their wild-type (WT) at 7 weeks of age were individually housed in plastic cages with a 12-h light:12-h dark cycle. To examine the effects of FGF21 on skeletal muscle atrophy in obesity, mice were fed a high-fat diet (HFD) (60% of calories as fat from lard and soybean oil; Research Diets, New Brunswick, NJ, USA) for 12 weeks and were given free access to food and water. Animals were sacrificed by CO_2_ asphyxiation, and their muscles were dissected. All animal care and procedures were conducted according to the protocols and guidelines approved by the University of Ulsan Animal Care and Use Committee (LNY-16-020).

### NF-κB activity

NF-κB DNA binding activity was assessed using a NF-κB p65 TransAM kit (Active Motif, Rixensart, Belgium). Samples of tissue homogenates or myotubes normalized for protein content were incubated with immobilized oligonucleotides containing an NF-κB consensus binding site. DNA binding activity was analyzed with antibodies specific for the NF-κB subunits according to the manufacturer’s instructions (Active Motif).

### Real-time PCR analysis

Total RNA was extracted from myotubes or muscle tissue samples with Tri-reagent (Life Technologies, Carlsbad, CA, USA). Two microgram aliquots of total RNA were reverse transcribed to cDNA using M-MLV reverse transcriptase (Promega, Madison, WI, USA). Real-time PCR amplification of cDNA was performed using SYBR premix Ex Taq kit (TaKaRa Bio Inc., Foster, CA, USA) with a Thermal Cycler Dice (TaKaRa Bio Inc., Otsu, Siga, Japan). All reactions were performed according to the same schedule: 95 °C for 10 s, 45 cycles at 95 °C for 5 s, and 60 °C for 30 s. The results were analyzed using Real Time System TP800 software (TaKaRa Bio Inc.), and all values were normalized to levels of the housekeeping gene, β-actin. The primers used in the analysis are listed in Table 1.

**Table 1:** Mouse primers used for qRT-PCR

Gene	Forward primer (5′ → 3′)	Reverse primer (5′ → 3′)
TNFα	AAGCCTGTAGCCCACGTCGTA	GGCACCACTAGTTGGTTGTCTTTG
MCP-1	GCATCCACGTGTTGGCTCA	CTCCAGCCTACTCATTGGGATCA
Atrogin-1	ACATTCTGCCAGCTGCTGTTTC	TGAGTTGGATGCTGGGCCTAC
MuRF1	TGTCTCACGTGTGAGGTGCCTA	CACCAGCATGGAGATGCAGTTAC
ATF4	CGGCTGGTCGTCAACCTATAAAGTA	GGTAACTGTGGCGTTAGAGATCGT
CHOP	AGTGCATCTTCATACACCACCACA	CAGATCCTCATACCAGGCTTCCA
β-actin	CATCCGTAAAGACCTCTATGCCAAC	ATGGAGCCACCGATCCACA

### Western blot analysis

Tissues and cells were homogenized in lysis buffer containing 150 mM NaCl, 50 mM Tris-HCl, 1 mM EDTA, 50 mM NaF, 10 mM Na_4_P_2_O_7_, 1% IGEPAL, 2 mM Na_3_VO_4_, 0.25% protease inhibitor cocktail, and 1% phosphatase inhibitor cocktail. Homogenates were centrifuged at 12,000 g for 20 min at 4 °C. Samples containing 10~100 μg of total proteins were subjected to Western blot analysis using polyclonal antibodies specific for MuRF1 (1:1000, #sc-32,920, Santa Cruz Biotechnology, Santa Cruz, CA, USA), Atrogin-1 (1:1000, #AP2041, ECM Biosciences, Versailles, Kentucky, USA), phosphorylated-eukaryotic inhibition factor 2 α (eIF2α, 1:1000, #3597, Cell Signaling Technology, Danvers, MA, USA), eIF2α (1:1000, #9722, Cell Signaling), phosphorylated-protein kinase RNA-like endoplasmic reticulum kinase (PERK, 1:1000, #12814, Signalway Antibody, College Park, MD, USA), PERK (1:1000, #3192, Cell Signaling), phosphorylated-AMPK (1:1000, #2531, Cell Signaling), AMPK (1:1000, #2332, Cell Signaling), and α-tubulin (1:5000, ab7291, Abcam, Cambridge, MA, USA). Protein bands were detected using an enhanced chemiluminescence kit (PerkinElmer, Waltham, MA, USA) and was estimated using ImageQuant LAS4000 (GE Healthcare). Protein intensities were quantified by densitometry using Image J software.

### Histological analysis

Skeletal muscle tissues (quadriceps) were fixed overnight at room temperature in 10% formaldehyde and embedded in paraffin. Tissues were sectioned (8-μm thick), stained with hematoxylin-eosin (H&E), mounted on glass slides. Stained sections were observed with an Axio-Star Plus microscope (Carl Zeiss, Gottingen, Germany). The diameters of the muscle fibers were determined using microscope AxioVision software. Four microscopic fields for samples were counted at 200x magnification.

### Statistical analysis

The results are presented as the mean ± SEM of values obtained from repeated experiments. All experiments were repeated 3–4 times. Statistical analysis was performed using Student’s t-test or one-way ANOVA (analysis of variance) followed by Newman-Keuls multiple comparison test with Prism 5.0 software (Graphic Pad, San Diego, CA, USA). Differences were considered to be significant at *P* < 0.05.

## Results

### Knockout of FGF21 induces atrophic responses in the skeletal muscle of HFD-fed mice

**Fig. 1:**
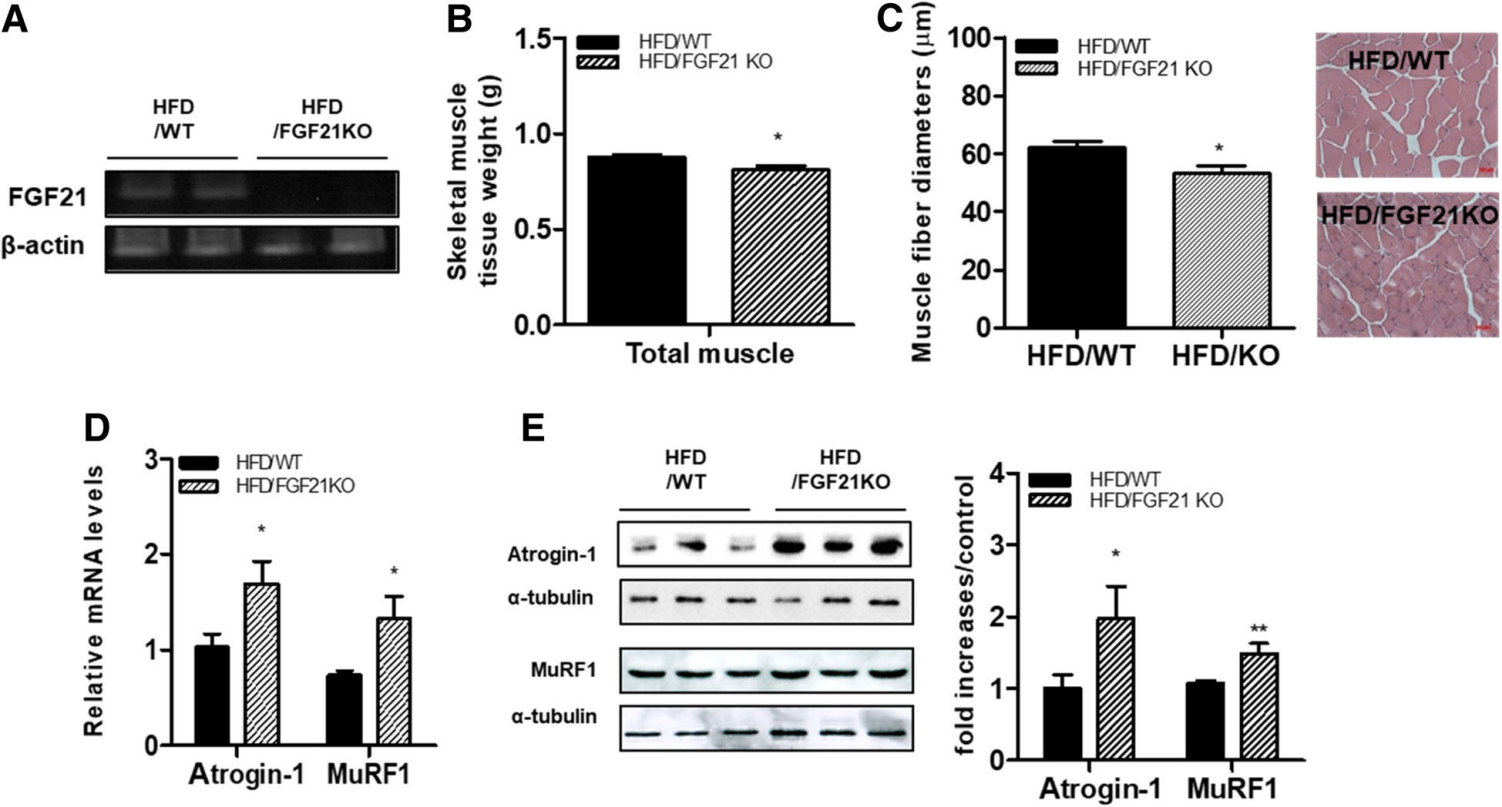
Effect of FGF21 deficiency on muscle weights and expression of atrophic factor-related genes/proteins in skeletal muscle of HFD-fed obese mice. FGF21-deficient and WT mice were fed an HFD for 12 weeks. (**a**) Expression of FGF21 mRNA in skeletal muscle. (**b**) Total muscle tissue weight (gastrocnemius, quadriceps, tibialis anterior, and soleus) from the experimental mice. (**c**) Histological analysis of quadriceps and mean diameters of muscle fibers from WT and FGF21-deficient mice fed an HFD. Sections were stained with hematoxylin and eosin. The diameters were determined with microscope AxioVision software. Magnification is × 200, Scale bar; 50 μm. The levels of muscle atrophic marker (Atrogin-1, MuRF1) (**d**) mRNA and (**e**) proteins in skeletal muscle. Results are means ± SEM (*n* = 4 mice per group). *P < 0.05, ** *P* < 0.01 compared with WT mice fed an HFD

In this study, we examined whether absence of FGF21 induces an obesity-induced atrophic response in skeletal muscle. We first confirmed that FGF21 mRNA was absent from the skeletal muscle of FGF21-deficient mice (Fig. 1a). The body weights of the FGF21-deficient mice given HFD were no different than those of the HFD-fed WT mice (HFD/WT: 44.75 ± 1.65 g, HFD/FGF KO: 45.61 ± 1.09 g). The weights of the skeletal muscle tissue were lower in the HFD-fed FGF21-deficient mice than in the HFD-fed mice (Fig. 1b). Histological examination of quadriceps cross sections showed that mean muscle fiber diameter was smaller in HFD-fed FGF21-deficient mice than HFD-fed WT mice (Fig. 1c). Next, we determined atrophic responses in the skeletal muscle of WT and FGF21-deficent mice fed an HFD. As shown in Fig. 1d, transcript levels of atrophic genes such as MuRF1 and Atrogin-1 were upregulated in the skeletal muscle of the HFD-fed FGF21-deficient mice compared to that of the HFD-fed WT mice. Western blot analysis revealed that the levels of atrophic proteins (MuRF1 and Atrogin-1) were increased in the HFD-fed FGF21-deficient mice compared to the HFD-fed WT mice (Fig. 1e).

### Knockout of FGF21 induces inflammation and endoplasmic reticulum (ER) stress in the skeletal muscle of HFD-fed mice

**Fig. 2:**
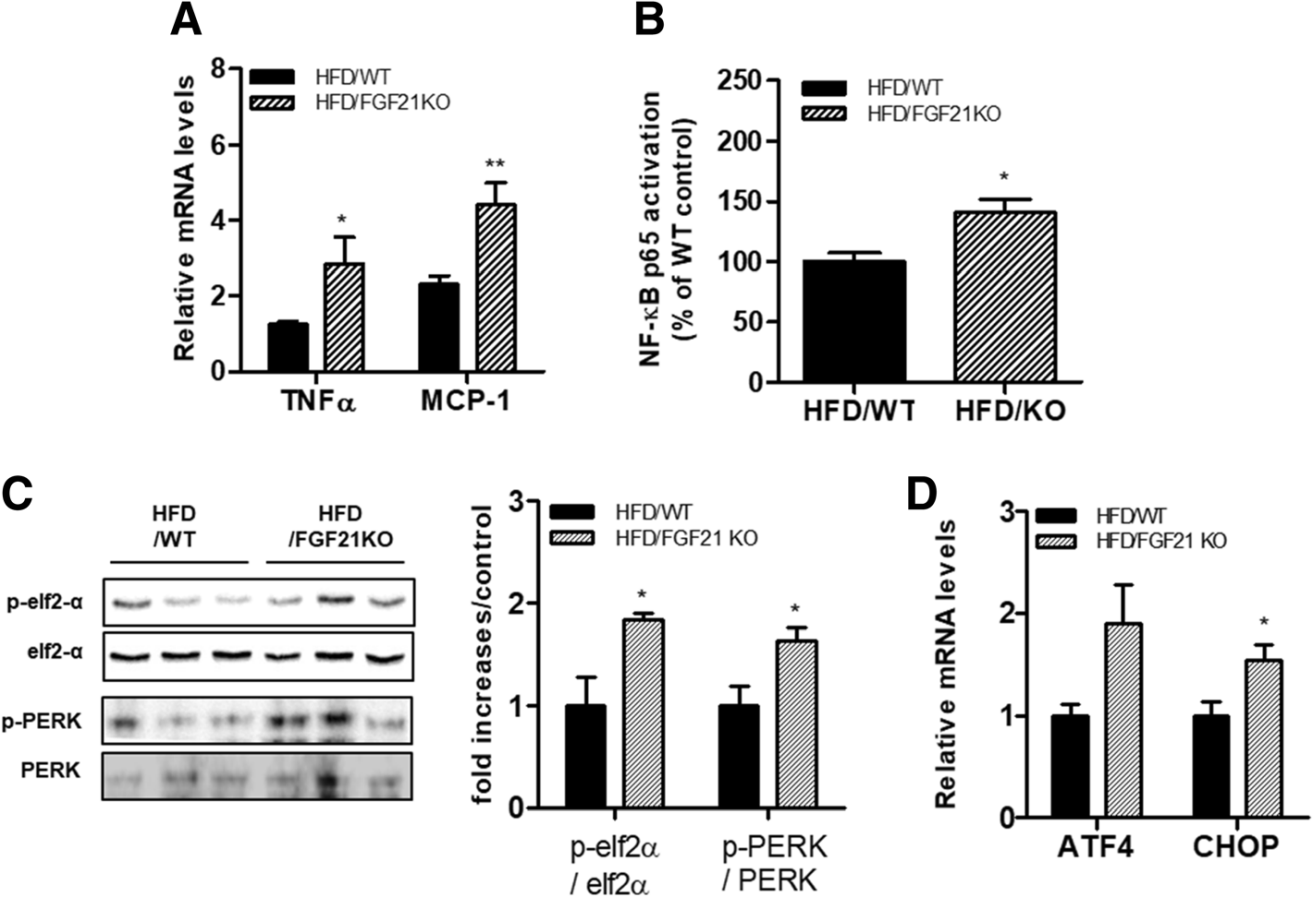
Effect of FGF21 deficiency on inflammatory responses in skeletal muscle of HFD-fed obese mice. (**a**) The levels of inflammatory cytokine (TNFα, MCP-1) mRNA in skeletal muscle from WT and FGF21-deficient mice fed an HFD. (**b**) NF-κB activation in skeletal muscle. NF-κB activation was determined using the p65 TransAM assay. (**c**) The levels of p-eIF2α/eIF2α and p-PERK/PERK protein in skeletal muscle. (**d**) The levels of activating transcription factor4 (ATF4) and CHOP mRNA in skeletal muscle. Results are means ± SEM (*n* = 4 mice per group). *P < 0.05, **P < 0.01 compared with WT mice fed an HFD

Next, we determined the effect of FGF21 deficiency on inflammatory responses in HFD-obese mice. Expression levels of inflammatory cytokines were markedly upregulated in the skeletal muscle of the HFD-fed FGF21-deficient mice compared with those of HFD-fed WT mice (Fig. 2a). Subsequently, we confirmed that the inflammatory signaling molecule estimated by activity of the NF-κB subunit p65 is enhanced in the skeletal muscle of the HFD-fed FGF21-deficient mice (Fig. 2b). Along with this, we observed levels of several ER stress markers (p-eIF2α and p-PERK) and mRNA expression of CCAAT-enhancer-binding protein homologous protein (CHOP) that were increased in the skeletal muscle of the HFD-fed FGF21-deficient mice compared with that of HFD-fed WT mice (Fig. 2c-d).

### Effects of FGF21 on TNFα-induced atrophic responses in myotubes

**Fig. 3:**
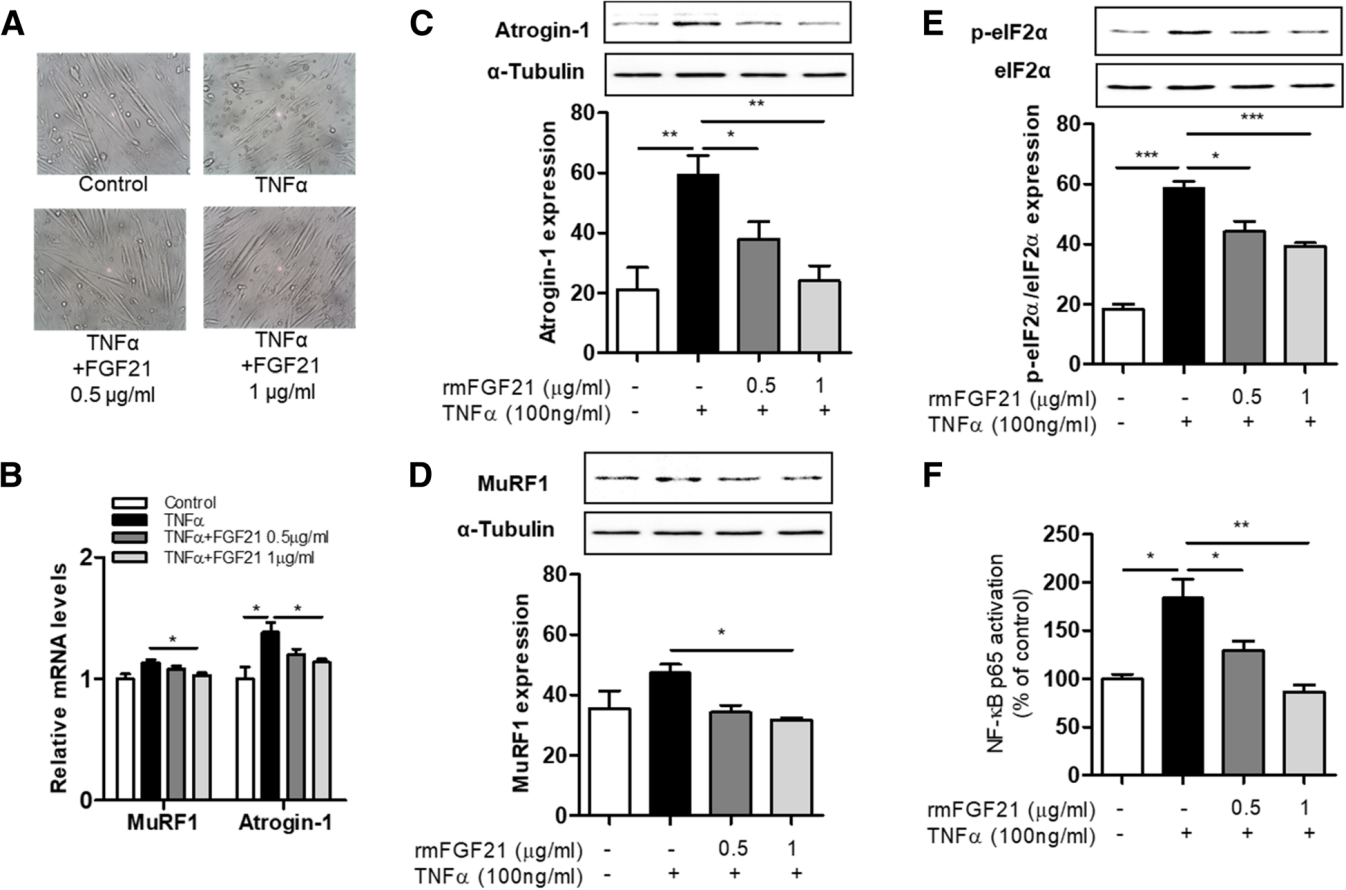
FGF21 suppresses the expression of atrophic factors and NF-kB activation in TNFα-treated myotubes. Myotubes at 2 days of differentiation were treated with 100 ng/mL TNFα in the presence or absence of FGF21 for 24 h. (**a**) Myotube morphology was observed at 100x magnification. The levels of (**b**) mRNA and (**c**-**d**) protein for MuRF1 and Atrogin-1 in C2C12 myotubes. (**e**) The levels of p-eIF2α/eIF2α protein in C2C12 myotubes. (**f**) NF-κB activation in C2C12 myotubes. Results are means ± SEM *P < 0.05, **P < 0.01, ***P < 0.001 compared with TNFα-treated myotubes

TNFα production is elevated in obesity-induced inflammatory states and is implicated as a major mediator of muscle atrophy. Here, we first examined the effect of FGF21 treatment on TNFα-induced muscle atrophy in C2C12 myotubes by analyzing changes in the morphological appearance of TNFα-treated C2C12 myotubes. TNFα treatment significantly reduced myotube diameter (Fig. 3a), whereas FGF21 treatment completely restored TNFα-induced reduction of myotube diameter (Fig. 3a). Subsequently, we examined whether or not FGF21 alters the expression of Atrogin-1 and MuRF1, which are markers of muscle atrophy. For this, myotubes were treated with TNFα, and atrophic markers were determined by RT-PCR and/or Western blotting analyses. FGF21 treatment significantly downregulated transcription levels of Atrogin-1 and MuRF1 (Fig. 3b) as well as reduced protein expression levels of Atrogin-1 and MuRF1 in TNFα-treated C2C12 myotubes (Fig. 3c and d). We next examined whether treatment of FGF21 on TNFα-stimulated C2C12 myotubes affected the ER stress and NF-κB signaling pathways. FGF21 treatment of TNFα-treated C2C12 myotubes led to decreased phosphorylation of eIF2α (Fig. 3e) and inactivation of NF-κB (Fig. 3f).

### rmFGF21 activated AMPK in C2C12 cells and skeletal muscle of HFD-fed obese mice

**Fig. 4:**
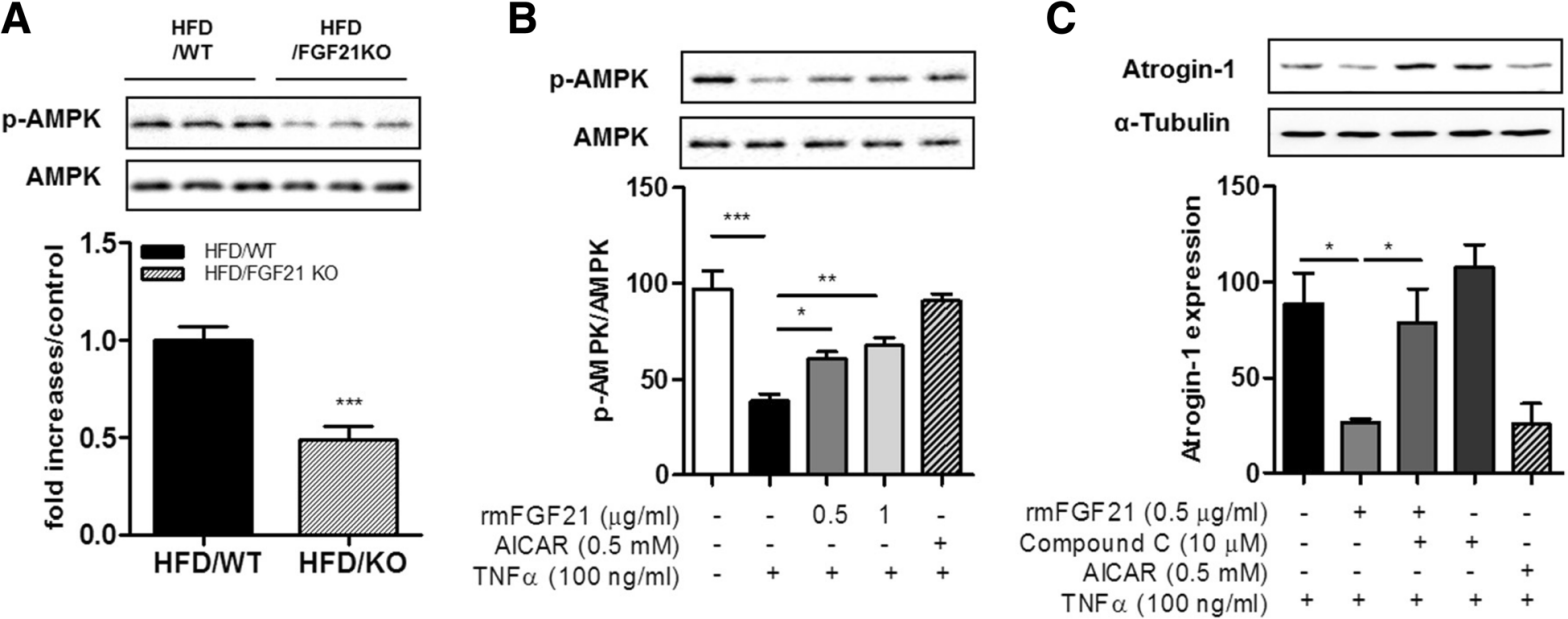
FGF21 reduces atrophic marker expression via AMPK pathway in TNFα–treated myotubes. (**a**) The levels of p-AMPK/AMPK protein in skeletal muscle from WT and FGF21-deficient mice fed an HFD. Results are means ± SEM (n = 4 mice per group). ***P < 0.001 compared with WT mice fed an HFD. (**b**) The levels of p-AMPK/AMPK protein in C2C12 myotubes were measured at the indicated concentrations (0, 0.5, 1 μg/mL) of FGF21 for 24 h. Results are means ± SEM *P < 0.05,**P < 0.01, ***P < 0.001 compared with TNFα-treated myotubes. (**c**) The levels of Atrogin-1 protein in TNFα-treated C2C12 myotubes with or without 0.5 μg/mL FGF21. Compound C added for 1 h before FGF21 treatment. Results are means ± SEM *P < 0.05 compared with TNFα plus FGF21-treated myotubes

To confirm whether the effect of FGF21 on obesity-induced skeletal muscle atrophy was related to AMPK, we investigated the AMPK phosphorylation by Western blotting. FGF21 deficiency significantly decreased AMPK phosphorylation in the muscle of HFD-fed mice (Fig. 4a). Incubation of C2C12 myotubes with rmFGF21 resulted in a dose-dependent increase in AMPK phosphorylation (Fig. 4b). Furthermore, we investigated whether AMPK is responsible for mediating the effect of rmFGF21. The AMPK inhibitor compound C suppressed the inhibitory actions of rmFGF21 on TNFα-induced atrophic responses in myotubes (Fig. 4c). This result indicates that the FGF21 action is mediated through AMPK activation.

## Discussion

Obesity-induced skeletal muscle atrophy is closely linked to inflammation and the development of metabolic disorders, and hence finding a new molecule linking skeletal muscle inflammation and atrophy may be beneficial to protect from obesity-related metabolic disorders. Previous studies have shown that endogenous levels of FGF21 increase in genetically and/or diet-induced obese mice, and the exogenous FGF21 response is severely impaired in the obese mice, suggesting that obesity is a state of FGF21 resistance [21, 22]. FGF21 protein is highly expressed in skeletal muscle [23], although expression level of the FGF21 co-receptor (β-Klotho) is low [24]. Given that muscle dependent systemic release of FGF21 also increases with obesity and ER stress [24], we hypothesized that FGF21 has the potential to protect obesity-induced atrophic responses. In this study, using FGF21-deficient mice fed an HFD, we showed that levels of atrophic markers such as MuRF1 and Atrogin-1 were markedly increased at the transcript and protein levels in FGF21-deficient obese mice, implicating that deficiency of FGF21 aggravates obesity-induced skeletal muscle atrophy. Of note, FGF21 has been shown to reduce levels of inflammatory mediators including IL-1β, IL-6 and TNFα in the serum of obese/diabetic db/db mice and ameliorates hyperglycemia [25]. Thus, we thought that the protective action of FGF21 against obesity-induced atrophic responses may be associated with its anti-inflammatory action. Indeed, we found that levels of inflammatory cytokines (TNFα and MCP-1) increased in the FGF21-deficient obese mice compared with WT control obese mice, and moreover in vitro treatment of myotubes with FGF21 protected against TNFα-induced atrophic responses. Taken together, these findings suggest that FGF21 has a protective role against inflammation-mediated skeletal muscle atrophy in obese conditions.

Obesity-induced inflammation is postulated to be a major contributor to skeletal muscle atrophy and metabolic dysregulation. TNFα, a potent NF-κB activator, is closely linked to loss of muscle mass in various inflammatory conditions such as obesity [26, 27]. TNFα and NF-κB in skeletal muscle enhances expression of ubiquitin ligases such as MuRF-1 and MAFbx/Atrogin-1 as well as inhibits regeneration of myofibers, supporting the link between muscle inflammation and atrophy. We observed that the deficiency of FGF21 enhanced production of inflammatory cytokines (TNFα and MCP-1) and activation of the NF-κB pathway in the skeletal muscle of obese mice compared with WT obese mice. This indicated that FGF21 deficiency augmented obesity-induced skeletal muscle inflammation and hence may exaggerate the ubiquitin-proteasome pathway, which is activated by the inflammatory signal, leading to increased protein degradation. Of note, ER stress mediates the activation of inflammatory signaling pathways such as the NF-κB pathway [28] and directly participates in reducing protein synthesis [25]. The ER stress transducer PERK phosphorylates eIF2α, and thereby reduces general protein synthesis [29], and TNFα is known to inhibit protein synthesis through increased phosphorylation of eIF2α [30]. Indeed, we found that the phosphorylation of eIF2α and other ER stress markers such as p-PERK and CHOP were markedly increased in FGF21-deficient obese mice compared with WT control obese mice. Taken together, the absence of FGF21 may enhance inflammatory signals that increase ER stress, and this may augment protein degradation and also impairs protein synthesis, further accelerating skeletal muscle atrophy in the FGF21-deficient obese mice.

Inflammation-induced AMPK activation suppresses anabolic signaling [31] and increases the expression of atrophy-related ubiquitin ligases [32], leading to muscle wasting [33]. However, AMPK has a potent anti-inflammatory effect [34], suggesting that AMPK activation could be beneficial to protect inflammation-associated muscle atrophic response. Indeed, studies have shown that a loss of AMPK accelerates aging-induced myopathy and mitochondrial dysfunction [35], and AMPK activation stimulates autophagy and ameliorates muscular dystrophy [36], FGF21 promotes myogenic differentiation and transformation of aerobic myofibers [37] via the AMPK pathway [38], and TNFα is known to inhibit AMP-activated protein kinase, leading to the reduction of oxidative capacity in skeletal muscle [39]. A recent study has demonstrated that the AMPK agonist AICAR suppresses inflammatory cytokines-induced muscle atrophy [40], which is consistent with our finding. More importantly, we observed that treatment with FGF21 increased the activation of AMPK with a concomitant decrease in Atrogin-1 in TNFα-treated C2C12 myotubes, which was antagonized by compound C, an AMPK inhibitor. Together, these findings suggest that FGF21 protects against the TNFα-induced atrophic response through AMPK activation, and thus the increased atrophic response in the skeletal muscle of FGF21-deficient obese mice may be associated with AMPK inactivation.

## Conclusion

In conclusion, we demonstrate that FGF21 deficiency aggravates the obesity-induced skeletal muscle inflammation and atrophy in the HFD-fed obese mice, accompanied by NF-κB activation and suppression of AMPK phosphorylation. FGF21 treatment in muscle cells protects against TNFα-induce atrophic responses through the AMPK pathway. FGF21 may be a potential target in combating obesity-related skeletal muscle atrophy.

## Data Availability

All data generated or analyzed during this study are included in this published article.

## Abbreviations

AMPK: AMP-activated protein kinase
eIF2α: Eukaryotic inhibition factor 2 α
ER: Endoplasmic reticulum
FGF21: Fibroblast growth factor 21
HFD: High-fat diet
IL-6: Interleukin-6
MAFbx: Muscle atrophy F-box
MuRF1: Muscle RING finger 1
NF-κB: Nuclear factor-kappa B
TNFα: Tumor necrosis factor α
WT: Wild-type
